# Diabetes Awareness and Acceptance in Patients with Type 2 Diabetes: A Single-Center Cross-Sectional Study in Istanbul, Türkiye

**DOI:** 10.3390/healthcare14172758

**Published:** 2026-08-31

**Authors:** Hatice Tursun, Arzu Erkoç

**Affiliations:** 1 Department of Nursing, Graduate Education Institute, Istanbul Sabahattin Zaim University, Istanbul 34303, Türkiye; hatice.tursunn@gmail.com; 2 Department of Internal Medicine Nursing, Faculty of Nursing, Istanbul University, Istanbul 34116, Türkiye

**Keywords:** acceptance, awareness, cross-sectional study, type 2 diabetes

## Abstract

**Highlights:**

**What are the main findings?**

- The mean DAAS total score among hospitalized adults with type 2 diabetes was 84.08 ± 12.02.
- Higher DAAS scores were independently associated with regular attendance at diabetes follow-up visits and adherence to dietary recommendations.

**What are the implications of the main findings?**

- Regular engagement with diabetes follow-up care was associated with higher diabetes awareness and acceptance.
- The findings identify regular follow-up attendance and dietary adherence as behavioral factors independently associated with higher diabetes awareness and acceptance.

**Abstract:**

**Objectives**: Diabetes continues to be a major and increasingly prevalent public health problem both globally and in Türkiye. Effective management of type 2 diabetes (T2D) largely depends on individuals’ ability to sustain daily self-management behaviors. This study was conducted to determine diabetes awareness and disease acceptance among hospitalized adults with T2D receiving care at a tertiary hospital in Istanbul, Türkiye, and to examine the sociodemographic and clinical factors associated with these levels. **Methods**: A descriptive, cross-sectional, and correlational design was used in accordance with the Strengthening the Reporting of Observational Studies in Epidemiology (STROBE) guidelines. The sample consisted of 118 patients selected using purposive consecutive sampling. Data were collected using the Diabetes Awareness and Acceptance Scale (DAAS). **Results**: The mean DAAS total score was 84.08 ± 12.02. Regular medication adherence, dietary adherence, and consistent attendance at diabetes follow-up visits were associated with higher awareness and acceptance levels. In the hierarchical multiple regression analysis, regular attendance at follow-up visits and dietary adherence remained independently associated with higher DAAS scores after adjustment for the other variables. **Conclusions**: This study provides descriptive evidence regarding diabetes awareness and acceptance among hospitalized adults with T2D. Higher DAAS scores were independently associated with regular attendance at diabetes follow-up visits and adherence to dietary recommendations. These findings indicate that engagement in recommended diabetes care behaviors is associated with diabetes awareness and acceptance. However, the findings should be interpreted in the context of hospitalized patients from a single tertiary-care center and may not be generalizable to all individuals with T2D in Türkiye.

## 1. Introduction

Diabetes continues to be a major and increasingly prevalent public health problem both globally and in Türkiye [1,2]. The growing disease burden indicates that diabetes is not merely a metabolic disorder but a multidimensional health condition with significant clinical, social, and economic consequences [3]. Therefore, diabetes remains a priority chronic condition that requires focused attention from both individuals and healthcare systems.

Effective management of Type 2 Diabetes (T2D) largely depends on individuals’ ability to sustain daily self-management behaviors. Behaviors such as adherence to medication, healthy eating, physical activity, blood glucose monitoring, and regular follow-up constitute the core components of diabetes management [4,5]. The sustainability of these behaviors plays a critical role in preventing complications and improving quality of life [6,7]. In this context, an individual’s level of knowledge and awareness regarding the disease is among the key determinants of developing effective self-care behaviors [8,9,10,11].

However, diabetes management is not limited to knowledge and awareness alone. Psychological acceptance of the disease is also an important factor for long-term treatment adherence and the sustainability of behavioral change. Awareness and acceptance are considered two complementary constructs. While awareness enables individuals to understand the seriousness of the disease and its management requirements, acceptance facilitates emotional adaptation and the internalization and maintenance of recommended treatment behaviors [12,13]. Therefore, awareness alone may not be sufficient, and acceptance may serve as a critical mechanism that translates knowledge into behavior.

Although the literature on diabetes education, self-management, and treatment adherence has been growing, studies that examine diabetes awareness and disease acceptance together remain limited [14,15,16]. Moreover, most existing studies have investigated the effects of these constructs separately, and evidence regarding their combined evaluation is insufficient. The limited number of studies addressing both concepts together in Türkiye highlights the need for further research at the national level. This gap is particularly important from a nursing perspective, as nurses play a key role in patient education, supporting behavioral change, and addressing the psychosocial needs of individuals with diabetes. It is well established that nurse-led self-management programs improve glycaemic control and that patient education enhances self-care behaviors [17]. Therefore, evaluating diabetes awareness and disease acceptance together may contribute to a more comprehensive understanding of patients’ adaptation to their illness and help identify target areas for nursing interventions.

This study was conducted to determine diabetes awareness and disease acceptance among hospitalized adults with T2D receiving care at a tertiary hospital in Istanbul, Türkiye, and to examine the sociodemographic and clinical factors associated with these levels. Accordingly, the following research questions were addressed:

- What are the levels of diabetes awareness and disease acceptance among individuals with T2D?
- Is there a significant relationship between the sociodemographic and clinical characteristics of individuals with T2D and their levels of diabetes awareness and disease acceptance?

## 2. Materials and Methods

### 2.1. Study Design and Data Collection

This study was designed as a descriptive, cross-sectional, and correlational study. The study was reported in accordance with the Strengthening the Reporting of Observational Studies in Epidemiology (STROBE) guidelines to ensure methodological rigor and transparency.

The study was conducted between November 2023 and June 2024 at a tertiary state hospital in Istanbul, Türkiye. The hospital is a 440-bed facility providing specialized care for patients with diabetes and other chronic diseases. Data were collected from four inpatient units: the Endocrinology and Metabolism Unit and three Internal Medicine Units.

The study population consisted of adult patients diagnosed with type 2 diabetes (T2D) who were hospitalized in these units during the study period. A purposive consecutive sampling strategy was employed. Patients receiving inpatient care were visited by the researcher three days per week between 16:00 and 20:00 throughout the study period. During each visit, all hospitalized patients who met the eligibility criteria were invited to participate. Recruitment continued until the end of the predefined data collection period, and a total of 118 eligible patients agreed to participate and completed the study.

Participants were eligible if they were 18 years of age or older, had been diagnosed with T2D for at least one year, were able to communicate effectively, and provided written informed consent. Patients who were unable to communicate because of severe cognitive impairment, acute medical instability, or other conditions preventing participation were not included.

Data were collected through face-to-face interviews conducted individually by the researcher in the patients’ rooms. Before data collection, each participant received information about the purpose of the study, the voluntary nature of participation, and the confidentiality of the collected data. Each interview lasted approximately 15–20 min.

### 2.2. Study Instruments

Data were collected using the Patient Information Form and the Diabetes Awareness and Acceptance Scale (DAAS). Patient Information Form: This form was developed by the researchers based on the literature [14,18]. It consists of two sections: (1) sociodemographic characteristics (age, gender, marital status, educational status, employment status, income status, living arrangement, and family history of T2D), and (2) clinical characteristics related to diabetes (duration of T2D, HbA1c, body mass index (BMI), type of treatment, regular medication adherence, regular physical activity, dietary adherence, and follow-up attendance). Medication adherence, physical activity, dietary adherence, and diabetes follow-up attendance were assessed using four single-item, self-reported questions with Yes/No response options. Participants were asked whether they regularly took their prescribed diabetes medications, engaged in physical activity as recommended by their healthcare provider, followed the dietary regimen recommended for diabetes management, and attended their scheduled diabetes follow-up visits. No validated adherence instrument, fixed retrospective reference period, or predefined quantitative threshold was used for these variables; therefore, ‘regular’ represented each participant’s self-reported usual behavior in relation to the recommendations or schedule provided by healthcare professionals. HbA1c values were obtained from the patients’ electronic medical records, and the most recent HbA1c measurement available during the index hospitalization was recorded. Height and weight were measured by ward nurses at hospital admission, and body mass index (BMI) was calculated by the researchers as weight in kilograms divided by height in meters squared (kg/m^2^). DAAS: The DAAS was developed by Atik et al. (2022) [18] to assess diabetes awareness and acceptance in patients with T2D. The scale consists of 23 items and two subdimensions: awareness (items 1–14) and acceptance (items 15–23). In the original scale development study, content validity, exploratory factor analysis (EFA), confirmatory factor analysis (CFA), and KMO and Bartlett’s tests supported a stable two-factor structure with satisfactory construct validity, explaining 79.5% of the total variance. The CFA demonstrated good model fit (GFI = 0.92, CFI = 0.97, RMSEA = 0.06), and the scale showed excellent internal consistency (Cronbach’s α = 0.967).

Each item is rated on a 5-point Likert scale ranging from 1 (never) to 5 (always). The total score of the scale ranges from 23 to 115. The awareness subscale ranges from 14 to 70, and the acceptance subscale ranges from 9 to 45. Higher scores obtained from the total scale and/or its subscales indicate higher levels of awareness and/or acceptance. The Cronbach’s alpha reliability coefficient of the original scale was reported as 0.967, with 0.945 for the awareness subscale and 0.948 for the acceptance subscale [18]. In the present study, the Cronbach’s alpha coefficient was found to be 0.892 for the total scale, 0.861 for the awareness subscale, and 0.909 for the acceptance subscale.

### 2.3. Statistical Analysis

Data were analyzed using IBM SPSS Statistics version 21.0 (IBM Corp., Armonk, NY, USA). Descriptive statistics were used to summarize the participants’ sociodemographic and clinical characteristics. The distribution of continuous variables was assessed using the Kolmogorov–Smirnov and Shapiro–Wilk tests, together with visual inspection of histograms and normal Q–Q plots. Normally distributed continuous variables were presented as mean ± standard deviation (SD), whereas non-normally distributed variables were summarized as median and interquartile range (IQR).

Between-group comparisons were performed according to the distribution of the data. For normally distributed continuous variables, the independent-samples *t*-test was used for comparisons between two groups and one-way analysis of variance (ANOVA) for comparisons among three or more groups. When the assumptions of normality were not satisfied, the Mann–Whitney U test and Kruskal–Wallis test were used for comparisons between two groups and three or more groups, respectively.

The internal consistency of the scale was assessed using Cronbach’s alpha coefficients. To identify factors independently associated with DAAS scores, multiple linear regression analysis with dummy-coded categorical variables was performed. All assumptions of regression analysis were evaluated. The Durbin–Watson statistic (1.791) indicated no violation of the independence of errors. In addition, no multicollinearity was detected (variance inflation factor < 5), and no influential outliers were identified (Cook’s distance < 1). A hierarchical multiple linear regression analysis was performed using the enter (block entry) method. Sociodemographic variables were entered in the first block, followed by diabetes-related clinical variables in the second block according to a predefined conceptual framework. A *p*-value of <0.05 was considered statistically significant.

## 3. Results

The study sample consisted of 118 patients with T2D. The mean total score on the DAAS was 84.08 ± 12.02. The mean subscale scores were 54.84 ± 7.24 for awareness and 29.15 ± 7.76 for acceptance (Table 1).

The mean age of the patients was 62.96 ± 12.6 years (range: 32–85), with more than half of the patients aged 65 years or younger (55.1%). The majority of the patients were female (55.1%) and married (75.4%). Most participants were literate (82.2%) and employed in income-generating occupations (87.3%). The majority were living with their spouse and children. Additionally, 22.9% of the patients reported a family history of T2D (Table 2).

Comparisons of DAAS scores according to the sociodemographic characteristics of the patients are presented in Table 3. No statistically significant differences were found in the total DAAS scores according to age, gender, marital status, employment status, income status, living arrangement, or family history of T2D (all *p* > 0.05). However, a statistically significant difference was observed in the acceptance subscale according to gender, with male patients demonstrating higher acceptance scores than female patients (Z = −2.06, *p* = 0.03). In addition, educational status was significantly associated with total DAAS scores, as literate patients had higher scores than illiterate patients (Z = −2.35, *p* = 0.01).

The mean duration of T2D among the patients was 12.38 ± 8.06 years (range: 1–40). The majority of the patients (67.8%) had been diagnosed with diabetes for 10 years or longer. While 60.2% of the patients had HbA1c levels above 7%, 72.9% had a BMI of 25 kg/m^2^ or higher. Regarding treatment modalities, 44.1% of the patients were receiving oral antidiabetic drugs (OAD) only, 33.9% were treated with insulin alone, and 22.0% were receiving combination therapy. Most patients (81.4%) reported regular medication adherence; however, only 14.4% engaged in regular physical activity, and 38.1% reported adherence to a recommended diet. Additionally, 63.6% of the patients reported regular attendance at diabetes follow-up visits (Table 2).

Comparisons of DAAS scores according to the clinical characteristics of the patients are presented in Table 4. No statistically significant differences in DAAS scores were found according to duration of T2D, HbA1c level, BMI, type of treatment, or regular physical activity (*p* > 0.05). However, a statistically significant difference was observed in the acceptance subscale according to type of treatment, with patients receiving OAD demonstrating higher acceptance scores compared with those treated with insulin alone or combination therapy (KW = 6.16, *p* = 0.04). Patients who reported regular medication adherence had significantly higher DAAS scores than those who did not adhere to their medication regimen (Z = −2.22, *p* = 0.02). In contrast, no statistically significant differences were found between medication adherence and awareness or acceptance subscale scores (*p* > 0.05).

Patients who adhered to a recommended diet had significantly higher DAAS and acceptance subscale scores than those who did not adhere to dietary recommendations (Z = −2.14, *p* = 0.03; Z = −2.12, *p* = 0.03, respectively). However, no statistically significant difference was observed in awareness subscale scores according to dietary adherence (*p* > 0.05). Furthermore, patients who regularly attended diabetes follow-up visits had significantly higher DAAS, awareness, and acceptance subscale scores than those who did not attend follow-up visits regularly (Z = −3.70, *p* < 0.001; Z = −2.70, *p* = 0.007; Z = −2.91, *p* = 0.004, respectively).

A hierarchical multiple linear regression analysis was performed to identify factors independently associated with DAAS scores (Table 5). The hierarchical multiple regression model was statistically significant (F = 3.266, *p* = 0.001). The addition of diabetes-related clinical variables in the second block increased the explained variance by 16.7% (ΔR^2^ = 0.167), resulting in a final model that explained 21.4% of the variance in DAAS scores (R^2^ = 0.214; adjusted R^2^ = 0.148). The standard error of the estimate was 11.095, and the Durbin–Watson statistic (1.834) indicated that the assumption of independence of residuals was met. Among the variables entered into the model, regular follow-up attendance (β = 0.332, *p* < 0.001) and dietary adherence (β = 0.178, *p* = 0.049) were independently associated with higher DAAS scores after adjustment for the other variables. None of the sociodemographic variables (age, gender, educational status, or income), duration of T2D, or regular medication adherence were significantly associated with DAAS scores after adjustment for the other variables (all *p* > 0.05). No evidence of multicollinearity was observed, with VIF values ranging from 1.078 to 1.255, and Cook’s distance (0.158) indicated that no influential observations unduly affected the regression model.

## 4. Discussion

In this study, diabetes awareness and disease acceptance were evaluated among adults with T2D using the DAAS. The total DAAS score provides an overall assessment of patients’ awareness and acceptance, whereas the awareness and acceptance subscales represent related but distinct dimensions. Therefore, the findings were interpreted using both the total DAAS score and the two subscale scores, together with their associations with sociodemographic and clinical characteristics.

The observed DAAS total, awareness, and acceptance scores indicate that the participants had developed a certain degree of understanding of their condition and had begun to incorporate diabetes into their daily lives, although important areas for further support remained. However, awareness of a diabetes diagnosis should not be assumed to result automatically in effective disease management. A recent systematic review and meta-analysis of population-representative studies showed that awareness and treatment rates were considerably higher than rates of adequate diabetes control, indicating a persistent gap between knowing about the condition, receiving treatment, and achieving desired clinical outcomes [16]. Similarly, a large international observational study found that self-management remained suboptimal and that approximately one-third of people with T2D reported treatment non-adherence, despite generally accepting the value of treatment [19]. These findings support the interpretation that diabetes awareness is necessary but may not be sufficient to ensure sustained treatment adherence and effective self-management.

Disease acceptance involves incorporating the illness and its requirements into daily life while reducing denial, resistance, and the emotional burden associated with living with a chronic condition. The acceptance scores observed in the present study suggest that some patients may still have experienced challenges in adapting their routines and behaviors to the demands of diabetes management. Recent evidence has demonstrated an association between illness acceptance and health-related behaviors among individuals with T2D, while also suggesting that psychological resources may contribute to this relationship [20]. Consequently, diabetes care should not be limited to transferring knowledge. Clinical assessment should also address patients’ illness perceptions, emotional responses, coping resources, motivation, and ability to incorporate recommended behaviors into everyday life.

The sociodemographic comparisons showed that total DAAS scores did not differ significantly according to age, gender, marital status, employment status, income, living arrangement, or family history of T2D. However, male participants had significantly higher acceptance scores than female participants, although their total DAAS and awareness scores were not significantly different. This finding should be interpreted specifically as a difference in the acceptance dimension rather than as evidence of an overall gender difference in diabetes awareness and acceptance. Because gender was not independently associated with the total DAAS score in the hierarchical regression model, the observed subscale difference may also reflect unmeasured psychological, social, caregiving, or disease-related factors. Further studies are needed to examine the potential mechanisms underlying gender differences in disease acceptance.

Literate participants had higher total DAAS scores than illiterate participants in the bivariate analysis. Literacy may facilitate access to diabetes-related information, communication with healthcare professionals, and understanding of treatment recommendations. However, educational status did not remain statistically significant after sociodemographic and clinical variables were considered simultaneously. Therefore, educational status should not be interpreted as being independently associated with DAAS scores in the present study. This finding may indicate that the relationship between educational status and DAAS scores partly overlaps with patients’ engagement in clinical follow-up and recommended self-management behaviors. It also underscores the importance of providing accessible, individualized, and health-literacy-sensitive education rather than assuming that the same educational approach will be equally effective for all patients.

Among the clinical characteristics, medication adherence, dietary adherence, and regular follow-up attendance were associated with higher total DAAS scores in the bivariate analyses. Acceptance scores also differed according to treatment type, and patients reporting dietary adherence had higher acceptance scores. Nevertheless, treatment type and medication adherence were not independently associated with total DAAS scores in the final regression model. These results illustrate the importance of distinguishing unadjusted group comparisons from independent associations obtained after simultaneous adjustment. Associations identified only in bivariate analyses may partly reflect confounding or shared variance with other clinical and behavioral characteristics.

In the hierarchical regression analysis, the addition of diabetes-related clinical variables to the sociodemographic variables increased the explained variance in total DAAS scores by 16.7%. The final model explained 21.4% of the variance, with regular follow-up attendance and dietary adherence independently associated with higher total DAAS scores. The substantially greater contribution of the clinical block suggests that engagement with recommended diabetes care behaviors may be more closely related to awareness and acceptance than the sociodemographic characteristics included in the model. However, the model left most of the variance unexplained, indicating that awareness and acceptance are multidimensional constructs that may also be influenced by health literacy, self-efficacy, illness perceptions, emotional distress, social support, patient–professional communication, and access to healthcare.

Regular follow-up attendance had the largest standardized regression coefficient and was therefore the strongest independent correlate of total DAAS scores among the variables included in the model. Regular clinical contact may provide repeated opportunities for education, evaluation of treatment barriers, reinforcement of self-management skills, and collaborative adjustment of care. Contemporary evidence supports the value of continuing diabetes self-management education and support rather than relying on a single educational encounter. A network meta-analysis reported that diabetes self-management education and support interventions produced clinically relevant improvements in glycemic and cardiovascular outcomes [8]. However, participation in structured education and follow-up remains challenging, and interventions intended to increase attendance do not necessarily improve all patient outcomes unless they successfully engage patients in the care process [21].

This association must not be interpreted as evidence that regular follow-up attendance caused higher diabetes awareness and acceptance. Because the present study was cross-sectional, the temporal sequence of the variables could not be established. Regular follow-up may provide education and support that contribute to greater awareness and acceptance; conversely, patients who already have greater awareness, acceptance, motivation, or confidence in managing diabetes may be more likely to attend scheduled visits. Both mechanisms may operate simultaneously. Prospective and longitudinal studies measuring awareness and acceptance before and after changes in follow-up participation are required to clarify the direction and potential reciprocity of this relationship.

Dietary adherence was also independently associated with higher total DAAS scores. Patients adhering to dietary recommendations had total DAAS scores approximately 4.4 points higher than those reporting non-adherence after adjustment for the other variables in the model. Awareness and acceptance may support dietary adherence by helping patients understand the purpose of dietary recommendations and incorporate them into their daily lives. Conversely, successfully adopting a dietary regimen may strengthen patients’ perceived control, engagement with diabetes care, and acceptance of the condition. A recent mixed-methods study showed that dietary adherence in T2D is influenced by interacting personal, social, cultural, and healthcare-related factors rather than knowledge alone [22]. Similarly, difficulties maintaining diabetes self-management behaviors after structured interventions have been linked to emotional, social, environmental, motivational, and practical barriers [23]. Dietary adherence should therefore be supported through individualized, culturally appropriate, feasible, and sustainable strategies rather than through information provision alone.

The combined findings have several implications for practice. Awareness and acceptance should be assessed as complementary components of diabetes management, particularly among patients who miss follow-up visits or experience difficulty following dietary recommendations. Follow-up encounters may be used to identify misunderstandings, emotional resistance, treatment burden, and practical obstacles to self-management. Education should be reinforced over time and adapted to patients’ literacy, readiness for behavioral change, daily routines, and available social resources. Recent randomized and systematic-review evidence indicates that structured, continuing, and technology-supported education can improve self-management behaviors, self-efficacy, and clinical outcomes among people with T2D [24,25,26]. Nevertheless, the present findings support only the identification of associated factors and do not establish that increasing follow-up attendance or dietary adherence would necessarily produce higher DAAS scores.

This study has several limitations. First, its cross-sectional and correlational design precludes conclusions regarding causality, directionality, or temporal sequence. In particular, although follow-up attendance and dietary adherence were independently associated with higher total DAAS scores, it cannot be determined whether these behaviors promote greater awareness and acceptance or whether individuals with greater awareness and acceptance are more likely to engage in these behaviors. Longitudinal and prospective studies are needed to investigate these potentially reciprocal relationships.

Second, the study was conducted at a single tertiary care hospital and included hospitalized patients recruited using non-probability consecutive sampling. Hospitalized individuals may have more severe or complex clinical conditions than patients managed in outpatient or primary care settings, resulting in potential selection bias. The findings should therefore not be generalized directly to all individuals with T2D, particularly those receiving care in primary healthcare facilities, rural communities, or different healthcare systems. Therefore, the findings should be interpreted as reflecting the characteristics of hospitalized adults with T2D receiving care at a single tertiary-care hospital in Istanbul rather than the broader population of people with T2D in Türkiye.

Third, medication adherence, dietary adherence, physical activity, and follow-up attendance were based on self-report and were measured as relatively broad categorical variables. These measures may be affected by recall and social desirability biases and may not capture the frequency, quality, duration, or consistency of the reported behaviors. Future studies should use validated multidimensional adherence instruments and, where feasible, objective measures such as appointment records, prescription-refill data, dietary assessments, activity monitoring, and clinical indicators.

Fourth, although several sociodemographic and clinical characteristics were included, potentially relevant psychological and contextual variables—including health literacy, diabetes-related distress, self-efficacy, illness perceptions, depression, social support, treatment burden, patient–professional communication, and access to healthcare—were not assessed. The final regression model explained 21.4% of the variance in DAAS scores, suggesting that these unmeasured factors may account for a substantial proportion of individual differences in diabetes awareness and acceptance.

Fifth, multiple exploratory bivariate comparisons were conducted without adjustment for multiple testing, which may have increased the probability of Type I error. Accordingly, isolated findings from the bivariate analyses, including differences in specific subscale scores, should be regarded as exploratory and interpreted cautiously until replicated. Greater emphasis should be placed on the adjusted hierarchical regression findings.

Finally, the relatively small sample and the number of variables entered into the regression model may have limited statistical precision and power to detect modest associations. The single-center design also reduced external validity. In addition, although the DAAS demonstrated good internal consistency in the present sample, its construct validity was not reassessed because this study was not designed as a psychometric validation study. Future multicenter studies with larger and more diverse samples should further examine the psychometric properties of the DAAS and evaluate prospective changes in awareness and acceptance.

## 5. Conclusions

This study showed that adults with T2D achieved a mean DAAS total score of 84.08 ± 12.02. Higher DAAS scores were independently associated with regular attendance at diabetes follow-up visits and adherence to dietary recommendations. These findings suggest that diabetes awareness and acceptance are related but distinct dimensions of living with diabetes and should therefore be assessed separately in clinical practice. Regular follow-up attendance and adherence to recommended self-management behaviors were associated with higher diabetes awareness and acceptance, although the direction of these relationships cannot be determined from the present cross-sectional study. However, these findings should be interpreted within the context of hospitalized patients from a single tertiary-care center and may not be generalizable to all individuals with T2D in Türkiye.

In the bivariate analyses, several sociodemographic and clinical characteristics were associated with total DAAS or subscale scores. However, after adjustment for the variables included in the hierarchical regression model, only regular follow-up attendance and dietary adherence remained independently associated with higher total DAAS scores. Regular follow-up attendance showed the strongest association among the variables included in the model. Nevertheless, because of the cross-sectional design, these findings should not be interpreted causally. It remains unclear whether regular follow-up attendance and dietary adherence contribute to greater diabetes awareness and acceptance or whether patients with higher awareness and acceptance are more likely to engage in these recommended care behaviors.

The final regression model explained 21.4% of the variance in DAAS scores, suggesting that additional psychological, social, behavioral, and healthcare-related factors may also be relevant to diabetes awareness and acceptance. Future studies should examine factors such as individualized education, continuity of follow-up, sustainable dietary behaviors, health literacy, self-efficacy, emotional adaptation, and other psychosocial needs in relation to diabetes awareness and acceptance. Longitudinal studies are needed to clarify the temporal direction of these relationships, while prospective interventional studies are required to determine whether modifying these factors can lead to changes in diabetes awareness and acceptance. Finally, the relatively small sample size and the number of variables entered into the regression model may have limited statistical precision and the ability to detect modest associations. These findings are specific to the study population and should not be generalized to the national population of individuals with T2D without confirmation from multicenter studies.

## Figures and Tables

**Table 1:** Diabetes awareness and acceptance scale scores of patients (*n* = 118).

Scale	Score Range	Min.–Max.	Median (Q1–Q3)	Mean (SD)
DAAS Total	23–115	26–106	85.5 (77–92)	84.08 (12.02)
Awareness	14–70	17–69	56.0 (51–59)	54.84 (7.24)
Acceptance	9–45	9–44	31.5 (24–36)	29.15 (7.76)

DAAS: diabetes awareness and acceptance scale; Max: maximum; Min: minimum; SD: standard deviation; Q1: 25th percentile; Q3: 75th percentile.

**Table 2:** Sociodemographic and clinical characteristics of patients (*n* = 118).

Variables	*n* (%)	Variables	*n* (%)
Age (years)		Duration of T2D (years)	
˂65	65 (55.1)	˂10	38 (32.2)
≥65	53 (44.9)	≥10	80 (67.8)
Gender		BMI	
Female	65 (55.1)	≥25	86 (72.9)
Male	53 (44.9)	˂25	32 (27.1)
Marital status		HbA1c (%)	
Single	29 (24.6)	≤7	47 (39.8)
Married	89 (75.4)	˃7	71 (60.2)
Educational status		Regular medication adherence	
Illiterate	21 (17.8)	Yes	96 (81.4)
Literate	97 (82.2)	No	22 (18.6)
Employment status		Regular physical activity	
Employed	103 (87.3)	Yes	17 (14.4)
Unemployed	15 (12.7)	No	101 (85.6)
Income status		Type of treatment	
Income < Expense	41 (34.8)	OAD	52 (44.1)
Income = Expense	70 (59.3)	Insulin	40 (33.9)
Income > Expense	7 (5.9)	Combination	26 (22)
Living arrangement		Dietary adherence	
Living alone	10 (8.5)	Yes	45 (38.1)
Living with spouse	30 (25.4)	No	73 (61.9)
Living with spouse and children	51 (43.2)	Follow-up attendance	
Living with other family members	15 (12.7)	Yes	75 (63.6)
Other	12 (10.2)	No	43 (36.4)
Family history of T2D			
Yes	27 (22.9)		
No	91 (77.1)		

BMI: body mass index, OAD: oral antidiabetic drugs, T2D: type 2 diabetes.

**Table 3:** DAAS scores according to sociodemographic characteristics (*n* = 118).

	DAAS	Test	Awareness	Test	Acceptance	Test
Variables	Median (Q1–Q3)	*p*-Values	Median (Q1–Q3)	*p*-Values	Median (Q1–Q3)	*p*-Values
Age (years)						
˂65	85.00 (78.50–92.00)	−0.39 ^Z^ 0.69	56.00 (53.00–59.00)	−1.15 ^Z^ 0.25	30.00 (24.50–36.00)	−0.04 ^Z^ 0.96
≥65	86.00 (76.50–91.50)		56.00 (50.50–58.50)		32.00 (21.50–36.00)	
Gender						
Female	84.00 (77.50–90.50)	−1.56 ^Z^ 0.11	56.00 (51.00–59.00)	−0.24 ^Z^ 0.80	29.00 (23.00–34.00)	−2.06 ^Z^ 0.03 *
Male	87.00 (76.50–93.50)		56.00 (51.00–58.00)		34.00 (26.00–36.00)	
Marital status						
Single	87.00 (78.50–92.50)	−1.70 ^Z^ 0.08	56.00 (53.00–59.00)	−1.67 ^Z^ 0.09	32.00 (24.00–36.00)	−0.85 ^Z^ 0.39
Married	83.00 (71.50–90.50)		56.00 (48.50–56.50)		29.00 (21.50–36.00)	
Educational status						
Illiterate	78.00 (68.50–90.00)	−2.35 ^Z^ 0.01 *	55.00 (45.00–57.50)	−1.63 ^Z^ 0.10	25.00 (19.50–34.00)	−1.73 ^Z^ 0.08
Literate	86.00 (80.00–92.50)		56.00 (52.50–59.00)		32.00 (26.00–36.00)	
Employment status						
Employed	87.00 (81.00–90.00)	−0.16 ^Z^ 0.87	54.00 (48.00–58.00)	−0.89 ^Z^ 0.37	32.00 (30.00–36.00)	−0.97 ^Z^ 0.33
Unemployed	85.00 (76.00–92.00)		56.00 (52.00–59.00)		31.00 (22.00–36.00)	
Income status						
Income < Expense	84.00 (75.50–92.00)	1.20 ^KW^ 0.54	56.00 (50.00–57.00)	1.85 ^KW^ 0.39	32.00 (21.00–36.00)	2.28 ^KW^ 0.31
Income = Expense	86.00 (77.75–92.00)		56.00 (51.00–59.00)		30.00 (24.00–36.00)	
Income > Expense	88.00 (83.00–96.00)		56.00 (53.00–56.00)		36.00 (31.00–36.00)	
Living arrangement						
Living alone	88.500 (81.00–93.25)	4.57 ^KW^ 0.33	56.00 (52.50–59.25)	6.28 ^KW^ 0.17	31.50 (28.00–36.25)	3.05 ^KW^ 0.54
Living with spouse	85.00 (74.75–91.25)		56.00 (50.00–59.50)		32.00 (18.00–35.25)	
Living with spouse and children	85.00 (78.00–92.00)		56.00 (53.00–59.00)		31.00 (24.00–36.00)	
Living with other family members	85.00 (76.00–93.00)		56.00 (51.00–57.00)		34.00 (24.00–36.00)	
Other	85.50 (73.00–90.75)		55.50 (51.25–56.75)		30.00 (21.00–35.50)	
Family history of T2D	Mean ± SD		Mean ± SD		Mean ± SD	
Yes	85.52 ± 11.63	0.21 ^t^ 0.82	54.15 ± 6.02	−0.56 ^t^ 0.57	30 ± 8.06	0.64 ^t^ 0.52
No	83.95 ± 12.19		55.04 ± 7.58		28.9 ± 7.7	

BMI: body mass index, DAAS: diabetes awareness and acceptance scale, OAD: oral antidiabetic drugs, T2D: type 2 diabetes, SD: standard deviation, Q1: 25th percentile, Q3: 75th percentile, ^KW^: Kruskal–Wallis test; ^t^: independent samples *t* test, ^Z^: Mann–Whitney U test, * *p* < 0.05.

**Table 4:** DAAS scores according to clinical characteristics (*n* = 118).

	DAAS	Test	Awareness	Test	Acceptance	Test
Variables		*p*-Values		*p*-Values		*p*-Values
Duration of T2D (years)	**Mean ± SD**		**Mean ± SD**		**Mean ± SD**	
˂10	85.08 ± 10.92	0.62 ^t^ 0.53	54.58 ± 6.59	−0.26 ^t^ 0.79	30.5 ± 6.54	1.41 ^t^ 0.16
≥10	83.6 ± 12.54		54.96 ± 7.56		28.51 ± 8.24	
BMI						
≥25	83.86 ± 12.17	−0.31 ^t^ 0.75	55.06 ± 7.44	0.53 ^t^ 0.59	28.69 ± 7.8	−1.07 ^t^ 0.28
˂25	84.66 ± 11.77		54.25 ± 6.74		30.41 ± 7.65	
HbA1c (%)	**Median (Q1** **–** **Q3)**		**Median (Q1** **–** **Q3)**		**Median (Q1** **–** **Q3)**	
≤7	86.00 (78.00–92.00)	−0.04 ^Z^ 0.96	56.00 (51.00–58.00)	−0.78 ^Z^ 0.43	32.00 (24.00–36.00)	−0.19 ^Z^ 0.84
˃7	85.00 (76.00–92.00)		56.00 (51.00–59.00)		30.00 (24.00–36.00)	
Type of treatment						
OAD	86.00 (78.50–93.00)	3.84 ^KW^ 0.14	56.00 (79.00–93.00)	3.21 ^KW^ 0.20	32.50 (26.00–36.00)	6.16 ^KW^ 0.04 *
Insulin	87.00 (76.50–92.75)		56.00 (77.00–92.50)		32.00 (22.50–36.00)	
Combination	83.00 (74.00–89.25)		56.00 (75.00–89.00)		28.00 (21.00–30.50)	
Regular medication adherence						
Yes	86.00 (79.00–92.75)	−2.22 ^Z^ 0.02 *	56.00 (52.00–59.00)	−1.59 ^Z^ 0.11	32.00 (25.00–36.00)	−1.78 ^Z^ 0.07
No	83.50 (69.00–88.25)		55.00 (47.25–58.00)		28.50 (19.75–33.00)	
Regular physical activity						
Yes	89.00 (79.00–93.50)	−1.19 ^Z^ 0.23	56.00 (49.50–59.50)	−0.27 ^Z^ 0.78	34.00 (27.50–36.00)	−1.56 ^Z^ 0.11
No	85.00 (76.00–91.00)		56.00 (51.50–59.00)		31.00 (22.00–36.00)	
Dietary adherence						
Yes	88.00 (82.50–93.50)	−2.14 ^Z^ 0.03 *	56.00 (53.00–59.50)	−1.20 ^Z^ 0.22	34.00 (26.00–36.00)	−2.12 ^Z^ 0.03 *
No	84.00 (75.00–91.00)		56.00 (51.00–58.00)		30.00 (21.00–35.50)	
Follow-up attendance						
Yes	87.00 (83.00–93.00)	−3.7 ^Z^ ˂0.001 **	56.00 (55.00–59.00)	−2.7 ^Z^ 0.007 **	32.00 (28.00–36.00)	−2.91 ^Z^ 0.004 **
No	80.00 (68.00–88.00)		53.00 (48.00–58.00)		27.00 (19.00–34.00)	

BMI: body mass index, DAAS: diabetes awareness and acceptance scale, OAD: oral antidiabetic drugs, T2D: type 2 diabetes, SD: standard deviation, Q1: 25th percentile, Q3: 75th percentile, ^KW^: Kruskal–Wallis test; ^t^: independent samples *t* test, ^Z^: Mann–Whitney U test, * *p* < 0.05, ** *p* < 0.01.

**Table 5:** Hierarchical multiple linear regression analysis predicting diabetes awareness and acceptance scale scores.

Model Summary	R	R^2^	Adj.R^2^	SE of the Estimate	F	*p*	DW
Model 1	0.216	0.047	0.004	12.000	1.093	0.368	-
Model 2	0.463	0.214	0.148	11.095	3.266	<0.001 *	1.834
Variables		Unstandardized Coefficients		Standardized Coefficients	*t*	*p* **	VIF
		B	SE	ꞵ			
Constant		72.897	3.755	-	19.414	<0.001	-
Independent Variables	Age (≥65)	0.171	2.262	0.007	0.075	0.940	1.214
	Gender (male)	2.406	2.265	0.100	1.062	0.290	1.216
	Educational status (Literate)	3.824	2.992	0.122	1.278	0.204	1.255
	Income status (Income = Expense)	−0.310	2.304	−0.013	−0.135	0.893	1.227
	Income status (Income ˃ Expense)	2.102	4.631	0.041	0.454	0.651	1.147
	Duration of T2D (≥10)	−3.046	2.270	−0.119	−1.342	0.182	1.078
	Regular medication adherence (Yes)	2.557	2.791	0.083	0.916	0.362	1.32
	Dietary adherence (Yes)	4.396	2.210	0.178	1.989	0.049	1.104
	Follow-up attendance (Yes)	8.255	2.247	0.332	3.674	<0.001	1.121
Dependent Variable:	Diabetes Awareness and Acceptance Scale					Cook’s: 0.158	

Adj.R^2^: Adjusted R2, Cook’s: Cook’s Distance, DAAS: Diabetes Awareness and Acceptance Scale, DW: Durbin–Watson, SE: Standard Error, VIF: Variance Inflation Factor, * Model significance based on one-way analysis of variance, ** Independent variable significance based on *t*-test.

## Data Availability

The data presented in this study are available on reasonable request from the corresponding author. The data are not publicly available due to ethical and privacy restrictions involving human participants.

## References

[1] Ceriello A., Colagiuri S. (2025). IDF global clinical practice recommendations for managing type 2 diabetes–2025. Diabetes Res. Clin. Pract..

[2] Rooney M.R., He J.H., Salpea P., Genitsaridi I., Magliano D.J., Boyko E.J., Wallace A.S., Fang M., Selvin E. (2025). Global and regional prediabetes prevalence: Updates for 2024 and projections for 2050. Diabetes Care.

[3] Genitsaridi I., Salpea P., Salim A., Sajjadi S.F., Tomic D., James S., Thirunavukkarasu S., Issaka A., Chen L., Basit A. (2026). 11th edition of the IDF Diabetes Atlas: Global, regional, and national diabetes prevalence estimates for 2024 and projections for 2050. Lancet Diabetes Endocrinol..

[4] Amerzadeh M., Kisomi Z.S., Senmar M., Khatooni M., Hosseinkhani Z., Bahrami M. (2024). Self-care behaviors, medication adherence status, and associated factors among elderly individuals with type 2 diabetes. Sci. Rep..

[5] Gyan K.F., Agyenim-Boateng E., Hutton-Mensah K.A., Opare-Addo P.A., Gyabaah S., Ofori E., Asamoah O.Y., Naabo M.N., Owiredu M.A., Tannor E.K. (2025). Predictors of glycemic control, quality of life and diabetes self-management of patients with diabetes mellitus at a tertiary hospital in Ghana. PLoS ONE.

[6] Bozkaya D.N., Gök Metin Z. (2023). An overview of the relationship between symptom status, self-management and care dependency in type 2 diabetes. J. Hacet. Univ. Fac. Nurs..

[7] American Diabetes Association Professional Practice Committee (2024). 5. Facilitating positive health behaviors and well-being to ımprove health outcomes: Standards of care in diabetes—2024. Diabetes Care.

[8] Romadlon D.S., Tu Y., Chen Y., Hasan F., Kurniawan R., Chiu H. (2024). Comparative effects of diabetes self-management programs on type 2 diabetes clinical outcomes: A systematic review and network meta-analysis. Diabetes/Metab. Res. Rev..

[9] Chowdhury H.A., Harrison C.L., Siddiquea B.N., Tissera S., Afroz A., Ali L., Joham A.E., Billah B. (2024). The effectiveness of diabetes self-management education intervention on glycaemic control and cardiometabolic risk in adults with type 2 diabetes in low-and middle-income countries: A systematic review and meta-analysis. PLoS ONE.

[10] Chen Y.C., Huang Y.H., Lee C.H. (2025). The Relationship Between Diabetes Knowledge and Diabetes Self-Care Behaviors in Relation to Diabetes Distress in Type 2 Diabetes Mellitus: A Cross-Sectional Study in Eastern Taiwan. Diabetes Metab. Syndr. Obes. Targets Ther..

[11] Ferreira P.L., Morais C., Pimenta R., Ribeiro I., Amorim I., Alves S.M., Santiago L. (2024). Knowledge about type 2 diabetes: Its impact for future management. Front. Public Health.

[12] Liu X., Zhang Q., Huang L., Zhuang Y. (2025). Associations between disease acceptance and dietary adherence in patients with type 2 diabetes mellitus in China: A cross-sectional study. Diabetes Res. Clin. Pract..

[13] Erdoğan Yüce G., Yıldırım H. (2025). Mediating role of disease acceptance in the relationship between social support and adherence to treatment in patients with type 2 diabetes. J. Behav. Med..

[14] Sendur U.G., Adas M. (2021). Determinants of awareness on diabetes and its complications. Exp. Clin. Endocrinol. Diabetes.

[15] Anwer I., Shahzad M., Ijaz N., Gill S.I., Shahzad A., Usman M. (2022). Awareness and understanding of diabetes complications among patients of 47 diabetes mellitus. Pak. J. Med. Health Sci..

[16] Shahrestanaki E., Khonsari N.M., Seif E., Baygi F., Ejtahed H.-S., Sheidaei A., Djalalinia S., Magliano D.J., Qorbani M. (2024). The worldwide trend in diabetes awareness, treatment, and control from 1985 to 2022: A systematic review and meta-analysis of 233 population-representative studies. Front. Public Health.

[17] Virtič Potočnik T., Mihevc M., Zavrnik Č., Lukančič M.M., Gorenjec N.R., Susič A.P., Klemenc-Ketiš Z. (2024). Evaluation of a specialist nurse-led structured self-management training for peer supporters with type 2 diabetes mellitus with or without comorbid hypertension in Slovenia. BMC Nurs..

[18] Atik D., Manav A.İ., Keser E. (2022). A scale development study: Diabetes awareness and acceptance scale. Diabetes Stoffwech. H..

[19] Chan J.C., Mbanya J.C., Chantelot J., Shestakova M., Ramachandran A., Ilkova H., Deplante L., Rollot M., Melas-Melt L., Gagliardino J.J. (2024). Patient-reported outcomes and treatment adherence in type 2 diabetes using natural language processing: Wave 8 of the Observational International Diabetes Management Practices Study. J. Diabetes Investig..

[20] Khazew H.R., Faraj R.K. (2024). Illness acceptance and its relationship to health-behaviors among patients with type 2 diabetes: A mediating role of self-hardiness. Curr. Probl. Cardiol..

[21] Davies M.J., Bodicoat D.H., Brennan A., Dixon S., Eborall H., Glab A., Gray L.J., Hadjiconstantinou M., Huddlestone L., Hudson N. (2024). Uptake of self-management education programmes for people with type 2 diabetes in primary care through the embedding package: A cluster randomised control trial and ethnographic study. BMC Prim. Care.

[22] Wilson D., Diji A.K.-A., Marfo R., Amoh P., Duodu P.A., Akyirem S., Gyamfi D., Asare H., Armah J., Enyan N.I.E. (2024). Dietary adherence among persons with type 2 diabetes: A concurrent mixed methods study. PLoS ONE.

[23] Carvalho M., Dunne P., Kwasnicka D., Byrne M., McSharry J. (2024). Barriers and enablers to maintaining self-management behaviours after attending a self-management support intervention for type 2 diabetes: A systematic review and qualitative evidence synthesis. Health Psychol. Rev..

[24] Alharbi T.A.F., Alhumaidi B., Alharbi M.N., Ngo A.D., Alasqah I., Alharbi H.F., Albagawi B. (2023). Diabetes education self-management intervention in improving self-efficacy for people with type 2 diabetes in the Gulf Cooperation Council countries: A systematic review. Diabetes Metab. Syndr. Clin. Res. Rev..

[25] Pai L.W., Hung C.T., Chen L.L., Lin R.L., Lockwood C. (2024). Efficacy of a health education technology program in improving adherence to self-management behaviors and quality of life among adults with type 2 diabetes: A randomized controlled trial. Prim. Care Diabetes.

[26] Tsokani S., Seitidis G., Christogiannis C., Kontouli K.-M., Nikolakopoulos S., Zevgiti S., Orrego C., Ballester M., Suñol R., Heijmans M. (2024). Exploring the Effectiveness of Self-Management Interventions in Type 2 Diabetes: A Systematic Review and Network Meta-Analysis. Healthcare.

